# The Importance of Patient Empowerment: A Clinical Case of Hereditary Angioedem

**DOI:** 10.7759/cureus.47644

**Published:** 2023-10-25

**Authors:** Guida Maria Santos, Filipa M Andrade, Catarina Marrana, Sara Gouveia

**Affiliations:** 1 Family Medicine, SESARAM EPERAM, Madeira Island, PRT

**Keywords:** treatment compliance, management, patient empowerment, hereditary angioedema, c1 inhibitor

## Abstract

Hereditary angioedema (HAE) is a rare condition characterized by recurrent episodes of angioedema without urticaria or pruritus. Untreated angioedema can cause significant work absenteeism and, in rare cases, be lethal due to laryngeal involvement and suffocation. The authors report a case of a patient with laryngeal involvement who was unaware of the severity of their condition. Effective medical training in patient empowerment is essential, and it is an irreplaceable element in healthcare, as it contributes to therapeutic success.

## Introduction

Hereditary angioedema (HAE) is a condition characterized by recurrent episodes of angioedema without hives or pruritus, which most commonly affects the skin and mucosa of the respiratory and gastrointestinal systems [1]. The pathophysiology involves dysfunction or absence of the C1 inhibitor (C1-INH). C1-INH is an acute-phase reactant responsible for the inhibition of bradykinin, a potent vasodilator that increases vascular permeability and causes edema. It is evident that mast cell mediators or histamine are absent in this process, rendering antihistamines, adrenaline, or corticosteroids ineffective [2].

There are two types of HAE, with type I accounting for 85% of cases. In type I, there is a reduced secretion of C1-INH, affecting both sexes equally [2].

Diagnosis is typically established during childhood due to the early onset of symptoms and is based on a suggestive clinical history, along with at least two complementary studies [2,3].

The first-line treatment involves the replacement of C1-INH with either human plasma-derived or recombinant human C1-INH [2,4].

## Case presentation

The clinical case concerns a 25-year-old, apparently healthy male who reported to the emergency department with complaints of odynophagia “similar to previous times” and hoarseness. According to the Manchester triage, he was classified as a green priority.

While collecting the medical history, it was found that the patient had a personal history of HAE type 1 (Table 1) and was non-compliant with therapy, having missed his last appointments and therefore lacking follow-up in recent years. Unused prescriptions of danazol were found in the prescription portal. When asked about possible triggers for the episode, he mentioned that he had undergone a dental extraction the previous week. He denied similar episodes in recent months. He was normotensive and, on physical examination, eupneic, without rashes or other abnormalities. However, inspection of the oropharynx revealed significant edema of the palate, uvula, and glottis.

**Table 1:** Laboratory analysis (May of 2012) C1-INH, C1 esterase inhibitor

	Serum levels values	Reference values
C1-INH	3 mg/dL	21-38 mg/dL
Functional C1-INH	25%	68-120%
C4	3.2 mg/dL	10-40 mg/dL

The immunology and allergy unit was contacted, and instructions were given to initiate a subcutaneous injection of icatibant 30 mg. Subsequent symptomatic improvement was observed, and he was kept under observation for about six hours. He was discharged with aminocaproic acid at a dose of 12 g per day and referred back to the immunology and allergy unit. Furthermore, the importance of treatment compliance was reinforced, and the nature of his disease and the potential severity of the condition were explained.

## Discussion

HAE is a rare condition where the diagnosis requires a high index of suspicion considering the described symptoms. When left untreated, this condition can be lethal. Therefore, it is important to educate patients about certain triggers for acute attacks, such as stress, dental procedures, local trauma, infections, sleep deprivation, consumption of certain foods, hormonal changes, and medications like angiotensin-converting enzyme inhibitors (ACE inhibitors), as well as the potential severity of this condition [3].

Although the edema is self-limited even without treatment, laryngeal involvement can lead to asphyxiation and become fatal. Laryngeal edema occurs in half of the patients with this condition during their lifetime, and it can occur alone or in association with edema of the tongue, lips, uvula, and soft palate [5]. The signs and symptoms associated with this condition include odynophagia, a feeling of “tightness in the throat,” cough, and voice changes such as hoarseness. In the past, about one-third of patients died due to asphyxia before effective therapies were available [6], making it a medical emergency. Therefore, raising awareness about the severity of this condition is crucial.

The therapeutic approach to HAE includes acute (on-demand) and prophylactic treatment. Acute treatment aims to relieve symptoms during a crisis, while prophylaxis aims to reduce the likelihood of edema in response to a trigger (short-term prophylaxis) or reduce the recurrence, severity, or duration of attacks (long-term prophylaxis). The most effective drugs for the treatment of acute attacks are C1-INH concentrate and icatibant, a bradykinin receptor antagonist [2]. In long-term prophylaxis, when indicated, available drugs include C1-INH concentrate, lanadelumab, berotralstat, or modified oral androgens such as stanozolol and danazol [2]. As for short-term prophylaxis (e.g., before dental extraction), C1-INH concentrate (agent of choice) can be used before the procedure, or modified oral androgens can be used before and after the procedure. In this case, considering a trigger such as dental extraction, it would be extremely important for the patient to be aware of the need for short-term prophylaxis [2].

**Figure 1:**
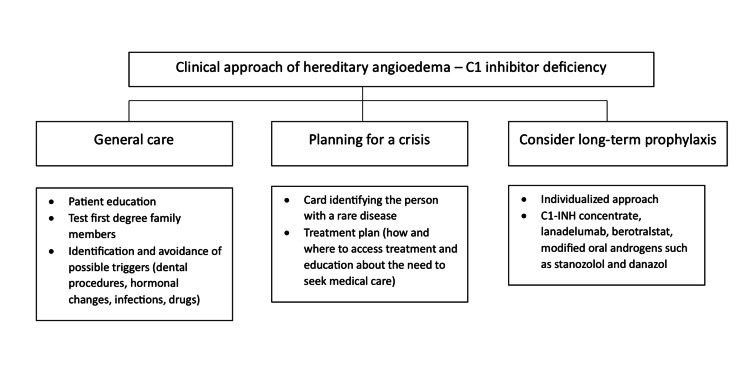
Clinical approach of hereditary angioedema: C1 inhibitor deficiency

The decision to initiate long-term prophylaxis is not based on strict criteria but should be individualized, taking into account aspects related to the patient, such as their needs, quality of life, disease, and available resources. This decision should be made with the patient. In Figure 1, we present a clinical approach of HAE.

In this clinical case, even though the patient was triaged as low priority, he had a potentially life-threatening condition that he did not identify. In an emergency setting, careful and immediate airway evaluation is paramount, as edema can progress rapidly, resulting in asphyxia and death.

Patient empowerment is a key element in healthcare quality. Patient empowerment is influenced by several factors, including patient-centered consultations. Patient-centered consultations are a comprehensive approach to healthcare that meets the patient’s individual needs and desires, not just their medical condition. They involve open communication and longer consultation times [7].

Most studies highlight the doctor-patient relationship, with the goal of developing good doctor-patient communication, as the primary predictor of patient empowerment [7-9].

In order to achieve better clinical outcomes, it is necessary to focus on the cognitive aspect of the patient, which involves empowering them with knowledge about their condition, teaching them potential triggers and how to efficiently control them, and identifying the necessary changes to be made in their life. In the medium to long term, the patient will be better equipped to manage the disease optimally, reducing the impact it may have on their life.

The prognosis for patients with HAE varies, as crises can occur repeatedly throughout their lives. Numbers regarding prognosis are limited, and the mortality rate appears to be around 13% when there is laryngeal involvement [10]. However, the frequency of these crises can be drastically reduced with treatment compliance.

## Conclusions

Regular follow-up of patients with HAE in the immunology and allergy unit is crucial. The aim of these follow-up visits is to assess the impact of the disease on the patient’s quality of life, disease control, and the need for short-term or long-term prophylactic treatment.

This clinical case highlights the importance of empowering patients with knowledge about their disease and how it can influence their management and control. It also emphasizes the role of patient empowerment in treatment compliance and the prevention of fatal outcomes. Patient empowerment is best achieved through patient-centered consultations that are built on a good doctor-patient relationship and effective communication.
